# Supplementary material for: ProteinShader: illustrative rendering of macromolecules
Source: BMC Struct Biol. 2009 Mar 30;9:19. doi: 10.1186/1472-6807-9-19 (PMC2672931; doi:10.1186/1472-6807-9-19)
Supplement: Additional file 1 — ProteinShader program without source code. This compressed file contains the complete ProteinShader program including associated libraries, but no source code. A README.txt file gives an overview of the ProteinShader distribution, and the index.html file in the help subdirectory has directions on getting started with the program as well as a set of tutorials. [file 1472-6807-9-19-S1.zip › ProteinShader-beta-0_9_4-binary/help/api/overview-tree.html]

Class Hierarchy (ProteinShader API)


|  |  |  |
| --- | --- | --- |
| **Overview** \| Package \| Class \| Use \| **Tree** \| **Deprecated** \| **Index** \| **Help** | |  |
| PREV   NEXT | **FRAMES**    **NO FRAMES**     **All Classes** |  |


---


## Hierarchy For All Packages

**Package Hierarchies:**: org.proteinshader.graphics, org.proteinshader.graphics.adapter, org.proteinshader.graphics.displaylists, org.proteinshader.graphics.enums, org.proteinshader.graphics.exceptions, org.proteinshader.graphics.shader, org.proteinshader.graphics.textures, org.proteinshader.graphics.typography, org.proteinshader.graphics.utils, org.proteinshader.gui, org.proteinshader.gui.components, org.proteinshader.gui.components.controlpanel, org.proteinshader.gui.components.menubar, org.proteinshader.gui.enums, org.proteinshader.gui.exceptions, org.proteinshader.gui.listeners, org.proteinshader.gui.listeners.controlpanel, org.proteinshader.gui.listeners.menubar, org.proteinshader.gui.utils, org.proteinshader.gui.viewing, org.proteinshader.math, org.proteinshader.structure, org.proteinshader.structure.enums, org.proteinshader.structure.exceptions, org.proteinshader.structure.factory, org.proteinshader.structure.io, org.proteinshader.structure.io.demos, org.proteinshader.structure.io.enums, org.proteinshader.structure.io.exceptions, org.proteinshader.structure.io.filters, org.proteinshader.structure.sort, org.proteinshader.structure.visitor, org.proteinshader.structure.visitor.demos, org.proteinshader.structure.visitor.enums, org.proteinshader.structure.visitor.exceptions, org.proteinshader.structure.visitor.modifiers

---

## Class Hierarchy

- java.lang.**Object**
  - org.proteinshader.gui.listeners.menubar.**AboutBoxListenerFactory**- org.proteinshader.gui.listeners.controlpanel.**AntialiasingPanelListenerFactory**- org.proteinshader.gui.listeners.menubar.**BackgroundMenuListenerFactory**- org.proteinshader.structure.io.**BetaStrandRecord**- org.proteinshader.structure.visitor.**BondPredictor**- org.proteinshader.gui.viewing.**Camera**- org.proteinshader.gui.listeners.**CanvasKeyListener** (implements java.awt.event.KeyListener)- org.proteinshader.gui.listeners.controlpanel.**CartoonSideChainsPanelListenerFactory**- org.proteinshader.structure.**Category**- org.proteinshader.structure.io.enums.**CategoryEnumDemo**- org.proteinshader.structure.**Chain** (implements org.proteinshader.structure.IDTest, org.proteinshader.structure.visitor.Visitable)- org.proteinshader.gui.listeners.controlpanel.**ColorPanelListenerFactory**- java.awt.**Component** (implements java.awt.image.ImageObserver, java.awt.MenuContainer, java.io.Serializable)
                            - java.awt.**Container**
                              - javax.swing.**JComponent** (implements java.io.Serializable)
                                - javax.swing.**AbstractButton** (implements java.awt.ItemSelectable, javax.swing.SwingConstants)
                                  - javax.swing.**JMenuItem** (implements javax.accessibility.Accessible, javax.swing.MenuElement)
                                    - javax.swing.**JMenu** (implements javax.accessibility.Accessible, javax.swing.MenuElement)
                                      - org.proteinshader.gui.components.menubar.**BackgroundMenu**- org.proteinshader.gui.components.menubar.**FileMenu**- org.proteinshader.gui.components.menubar.**HelpMenu**- org.proteinshader.gui.components.menubar.**OrientationMenu**- org.proteinshader.gui.components.menubar.**StyleMenu**- org.proteinshader.gui.components.menubar.**ToolsMenu**- org.proteinshader.gui.components.menubar.**VisibilityMenu**
                                  - javax.swing.**JFileChooser** (implements javax.accessibility.Accessible)
                                    - org.proteinshader.gui.utils.**ImageFileChooser**
                                    - javax.swing.**JLabel** (implements javax.accessibility.Accessible, javax.swing.SwingConstants)
                                      - javax.swing.**DefaultListCellRenderer** (implements javax.swing.ListCellRenderer, java.io.Serializable)
                                        - org.proteinshader.gui.utils.**CenteredListCellRenderer**- org.proteinshader.gui.utils.**PaddedListCellRenderer**- org.proteinshader.gui.utils.**SeparatorListCellRenderer**
                                      - javax.swing.**JMenuBar** (implements javax.accessibility.Accessible, javax.swing.MenuElement)
                                        - org.proteinshader.gui.components.menubar.**MainMenuBar**
                                        - javax.swing.**JPanel** (implements javax.accessibility.Accessible)
                                          - org.proteinshader.gui.components.controlpanel.**AntialiasingPanel**- org.proteinshader.gui.components.controlpanel.**AtomScalePanel**- org.proteinshader.gui.components.controlpanel.**CartoonSideChainsPanel**- org.proteinshader.gui.components.controlpanel.**ColorPanel**
                                                  - org.proteinshader.gui.components.controlpanel.**AtomColorPanel**- org.proteinshader.gui.components.controlpanel.**CartoonColorPanel**
                                                  - org.proteinshader.gui.components.controlpanel.**ControlPanel**- org.proteinshader.gui.components.controlpanel.**DecorationsPanel**- org.proteinshader.gui.components.controlpanel.**ModifierPanel**- org.proteinshader.gui.components.controlpanel.**MotionPanel**- org.proteinshader.gui.components.controlpanel.**OptimizePanel**- org.proteinshader.gui.components.controlpanel.**RadioPanel**- org.proteinshader.gui.components.controlpanel.**SelectorPanel**- org.proteinshader.gui.components.controlpanel.**VisibilityPanel**
                                                                  - org.proteinshader.gui.components.controlpanel.**AtomVisibilityPanel**- org.proteinshader.gui.components.controlpanel.**CartoonVisibilityPanel**
                                - java.awt.**Window** (implements javax.accessibility.Accessible)
                                  - java.awt.**Dialog**
                                    - javax.swing.**JDialog** (implements javax.accessibility.Accessible, javax.swing.RootPaneContainer, javax.swing.WindowConstants)
                                      - org.proteinshader.gui.components.menubar.**AboutBox**
                                    - java.awt.**Frame** (implements java.awt.MenuContainer)
                                      - javax.swing.**JFrame** (implements javax.accessibility.Accessible, javax.swing.RootPaneContainer, javax.swing.WindowConstants)
                                        - org.proteinshader.gui.**ProteinShaderGUI**- org.proteinshader.gui.components.**SpringLoadedJFrame** (implements java.awt.event.ComponentListener, java.awt.event.WindowListener)
                            - org.proteinshader.graphics.displaylists.**CylinderReferences**- org.proteinshader.gui.listeners.controlpanel.**DecorationsPanelListenerFactory**- org.proteinshader.structure.**Description**- org.proteinshader.structure.**Drawable** (implements org.proteinshader.structure.visitor.Visitable)
                                    - org.proteinshader.structure.**Atom** (implements org.proteinshader.structure.IDTest)- org.proteinshader.structure.**Bond**- org.proteinshader.structure.**Segment**
                                    - org.proteinshader.structure.visitor.modifiers.**DrawableModifier**
                                      - org.proteinshader.structure.visitor.modifiers.**AtomModifier**- org.proteinshader.structure.visitor.modifiers.**BondModifier**- org.proteinshader.structure.visitor.modifiers.**SegmentModifier**
                                      - org.proteinshader.structure.sort.**DrawableSorter**- org.proteinshader.graphics.textures.**EqualSignConfigReader** (implements org.proteinshader.graphics.textures.ConfigReader)- javax.swing.filechooser.**FileFilter**
                                            - org.proteinshader.gui.utils.**FileExtensionFilter**- org.proteinshader.structure.io.filters.**PDBFileFilter**
                                            - org.proteinshader.gui.listeners.menubar.**FileMenuListenerFactory**- org.proteinshader.gui.utils.**FpsLabel**- org.proteinshader.graphics.displaylists.**GeometricListInfo**
                                                  - org.proteinshader.graphics.displaylists.**CylinderListInfo**- org.proteinshader.graphics.displaylists.**SegmentListInfo**- org.proteinshader.graphics.displaylists.**SphereListInfo**
                                                  - org.proteinshader.graphics.typography.**GLF2GlyphSetFactory** (implements org.proteinshader.graphics.typography.GlyphSetFactory)- org.proteinshader.graphics.typography.**GLF2Header**- org.proteinshader.graphics.typography.**Glyph**- org.proteinshader.graphics.typography.**GlyphDemo**- org.proteinshader.graphics.typography.**GlyphSet**- org.proteinshader.graphics.typography.**GlyphSetFactoryFactory**- org.proteinshader.structure.io.**HelixRecord**- org.proteinshader.gui.listeners.menubar.**HelpMenuListenerFactory**- org.proteinshader.math.**Hermite**- org.proteinshader.math.**HermiteDemo**- org.proteinshader.gui.viewing.**Lighting**- org.proteinshader.math.**LocalFrame**- org.proteinshader.math.**LocalFrameDemo**- org.proteinshader.gui.**MediatorImpl** (implements org.proteinshader.gui.Mediator)- org.proteinshader.structure.**Model** (implements org.proteinshader.structure.IDTest, org.proteinshader.structure.visitor.Visitable)- org.proteinshader.gui.listeners.controlpanel.**ModifierPanelListenerFactory**- org.proteinshader.structure.visitor.demos.**ModifierVisitorDemo**- org.proteinshader.gui.listeners.controlpanel.**MotionPanelListenerFactory**- javax.swing.event.**MouseInputAdapter** (implements javax.swing.event.MouseInputListener)
                                                                                        - org.proteinshader.gui.listeners.**CanvasMouseListener** (implements java.awt.event.MouseWheelListener)
                                                                                        - org.proteinshader.graphics.textures.**NamePair**- org.proteinshader.gui.listeners.controlpanel.**OptimizePanelListenerFactory**- org.proteinshader.gui.listeners.menubar.**OrientationMenuListenerFactory**- org.proteinshader.structure.io.**PDBAtomFieldExtractor**- org.proteinshader.structure.io.**PDBBetaStrandFieldExtractor**- org.proteinshader.structure.io.**PDBConnectFieldExtractor**- org.proteinshader.structure.io.**PDBHelixFieldExtractor**- org.proteinshader.structure.io.**PDBLineParser**- org.proteinshader.structure.io.**PDBStructureReader** (implements org.proteinshader.structure.io.StructureReader)- org.proteinshader.structure.io.demos.**PDBStructureReaderDemo**- org.proteinshader.math.**Point3d** (implements java.lang.Cloneable)- org.proteinshader.gui.**ProteinShader**- org.proteinshader.gui.**ProteinShaderSplasher**- org.proteinshader.math.**Quaternion** (implements java.lang.Cloneable)- org.proteinshader.math.**QuaternionDemo**- org.proteinshader.gui.listeners.controlpanel.**RadioPanelListenerFactory**- org.proteinshader.structure.**Record**- org.proteinshader.structure.io.enums.**RecordEnumDemo**- org.proteinshader.structure.**Region** (implements org.proteinshader.structure.IDTest, org.proteinshader.structure.visitor.Visitable)
                                                                                                                              - org.proteinshader.structure.**BetaStrand**- org.proteinshader.structure.**Helix**- org.proteinshader.structure.**Loop**
                                                                                                                              - org.proteinshader.gui.**Renderer** (implements javax.media.opengl.GLEventListener)- org.proteinshader.structure.**Residue** (implements org.proteinshader.structure.IDTest, org.proteinshader.structure.visitor.Visitable)
                                                                                                                                  - org.proteinshader.structure.**AminoAcid**- org.proteinshader.structure.**Heterogen**- org.proteinshader.structure.**Water**
                                                                                                                                  - org.proteinshader.gui.viewing.**Rotation**- org.proteinshader.gui.listeners.controlpanel.**ScalePanelListenerFactory**- org.proteinshader.gui.viewing.**ScreenShot**- org.proteinshader.structure.io.demos.**SegmentDemo**- org.proteinshader.structure.factory.**SegmentFactory**
                                                                                                                                            - org.proteinshader.structure.factory.**BetaStrandSegmentFactory**
                                                                                                                                            - org.proteinshader.graphics.displaylists.**SegmentReferences**- org.proteinshader.graphics.utils.**SegmentRenderer**- org.proteinshader.gui.listeners.controlpanel.**SelectorPanelListenerFactory**- org.proteinshader.graphics.shader.**ShaderManager**- org.proteinshader.graphics.shader.**ShaderProgram**- org.proteinshader.graphics.shader.**ShaderProgramFactory**- org.proteinshader.graphics.**Shape**
                                                                                                                                                          - org.proteinshader.graphics.**Cylinder**- org.proteinshader.graphics.**ExtrudedShape**
                                                                                                                                                              - org.proteinshader.graphics.**FrenetFrames**- org.proteinshader.graphics.**Ribbon**- org.proteinshader.graphics.**Tube**
                                                                                                                                                              - org.proteinshader.graphics.**Sphere**
                                                                                                                                                          - org.proteinshader.graphics.**Silhouette**- org.proteinshader.math.**SlerpDemo**- org.proteinshader.graphics.displaylists.**SphereReferences**- org.proteinshader.structure.**Structure** (implements org.proteinshader.structure.IDTest, org.proteinshader.structure.visitor.Visitable)- org.proteinshader.graphics.adapter.**StructureToGraphics**- org.proteinshader.gui.listeners.menubar.**StyleMenuListenerFactory**- org.proteinshader.graphics.typography.**TextLabel**- org.proteinshader.graphics.typography.**TextLabelFactory**- org.proteinshader.graphics.typography.**TextLabelManager**- org.proteinshader.graphics.textures.**Texture** (implements java.lang.Comparable<T>)- org.proteinshader.graphics.textures.**TextureFactory**- org.proteinshader.graphics.textures.**TextureManager**- java.lang.**Throwable** (implements java.io.Serializable)
                                                                                                                                                                                    - java.lang.**Exception**
                                                                                                                                                                                      - org.proteinshader.structure.exceptions.**BondLengthException**- org.proteinshader.structure.exceptions.**ColorOutOfRangeException**- org.proteinshader.graphics.exceptions.**GlyphException**
                                                                                                                                                                                            - org.proteinshader.graphics.exceptions.**GlyphConfigException**- org.proteinshader.graphics.exceptions.**GlyphImageException**
                                                                                                                                                                                            - org.proteinshader.structure.exceptions.**InvalidIDException**- org.proteinshader.structure.exceptions.**InvalidRegionException**- org.proteinshader.structure.exceptions.**MissingAATypeException**- org.proteinshader.structure.exceptions.**MissingAtomTypeException**- org.proteinshader.structure.exceptions.**MissingHetNameException**- org.proteinshader.structure.io.exceptions.**PDBFieldExtractorException**- org.proteinshader.structure.io.exceptions.**PDBLineParserException**- org.proteinshader.gui.exceptions.**ScreenShotException**- org.proteinshader.structure.exceptions.**SegmentException**- org.proteinshader.graphics.exceptions.**ShaderException**- org.proteinshader.structure.io.exceptions.**StructureReaderException**- org.proteinshader.graphics.exceptions.**TextureException**- org.proteinshader.structure.visitor.exceptions.**VisitorException**
                                                                                                                                                                                                                      - org.proteinshader.structure.visitor.exceptions.**WriterVisitorException**
                                                                                                                                                                                    - org.proteinshader.gui.listeners.menubar.**ToolsMenuListenerFactory**- org.proteinshader.structure.io.**TubeVertices**- org.proteinshader.math.**Vec3d** (implements java.lang.Cloneable)- org.proteinshader.math.**VectorAndPointDemo**- org.proteinshader.gui.listeners.menubar.**VisibilityMenuListenerFactory**- org.proteinshader.gui.listeners.controlpanel.**VisibilityPanelListenerFactory**- org.proteinshader.structure.visitor.**Visitor**
                                                                                                                                                                                                  - org.proteinshader.structure.visitor.**AminoAcidLabelVisitor**- org.proteinshader.structure.visitor.**BondDestroyerVisitor**- org.proteinshader.structure.visitor.**BondGeneratorVisitor**- org.proteinshader.structure.visitor.**BoundsVisitor**- org.proteinshader.structure.visitor.**FrenetFrameGeneratorVisitor**- org.proteinshader.structure.visitor.**LoopGeneratorVisitor**- org.proteinshader.structure.visitor.**ModifierVisitor**- org.proteinshader.structure.visitor.**SFWriterVisitor**- org.proteinshader.structure.visitor.**VisibilityVisitor**
                                                                                                                                                                                                  - org.proteinshader.structure.visitor.demos.**VisitorDemo**- org.proteinshader.gui.listeners.**WindowListenerFactory**

## Interface Hierarchy

- org.proteinshader.graphics.textures.**ConfigReader**- org.proteinshader.graphics.typography.**GlyphSetFactory**- org.proteinshader.structure.**IDTest**- org.proteinshader.gui.**Mediator**- org.proteinshader.structure.io.**StructureReader**- org.proteinshader.structure.visitor.**Visitable**

## Enum Hierarchy

- java.lang.**Object**
  - java.lang.**Enum**<E> (implements java.lang.Comparable<T>, java.io.Serializable)
    - org.proteinshader.graphics.enums.**ShaderEnum**- org.proteinshader.gui.components.controlpanel.**RadioPanel.Type**- org.proteinshader.gui.enums.**JitterEnum**- org.proteinshader.gui.enums.**StyleEnum**- org.proteinshader.gui.enums.**ImageFormatEnum**- org.proteinshader.gui.enums.**RadioButtonEnum**- org.proteinshader.gui.enums.**OrientationEnum**- org.proteinshader.gui.listeners.**CanvasMouseListener.Mode**- org.proteinshader.structure.enums.**RegionEnum**- org.proteinshader.structure.enums.**HelixShapeEnum**- org.proteinshader.structure.enums.**ResidueEnum**- org.proteinshader.structure.enums.**DecorationEnum**- org.proteinshader.structure.enums.**AAColorEnum**- org.proteinshader.structure.enums.**HelixEnum**- org.proteinshader.structure.enums.**DrawableEnum**- org.proteinshader.structure.enums.**VisibilityEnum**- org.proteinshader.structure.enums.**BondEnum**- org.proteinshader.structure.enums.**SideChainEnum**- org.proteinshader.structure.enums.**AminoAcidEnum**- org.proteinshader.structure.enums.**AtomEnum**- org.proteinshader.structure.enums.**RegionColorEnum**- org.proteinshader.structure.enums.**CPKColorEnum**- org.proteinshader.structure.io.enums.**CategoryEnum**- org.proteinshader.structure.io.enums.**RecordEnum**- org.proteinshader.structure.visitor.enums.**AAPortionMode**- org.proteinshader.structure.visitor.enums.**ResidueMode**- org.proteinshader.structure.visitor.enums.**RegionMode**

---


|  |  |  |
| --- | --- | --- |
| **Overview** \| Package \| Class \| Use \| **Tree** \| **Deprecated** \| **Index** \| **Help** | |  |
| PREV   NEXT | **FRAMES**    **NO FRAMES**     **All Classes** |  |


---

# *Copyright © 2007-2008*
